# The Impact of Policy Measures on Human Mobility, COVID-19 Cases, and Mortality in the US: A Spatiotemporal Perspective

**DOI:** 10.3390/ijerph18030996

**Published:** 2021-01-23

**Authors:** Yun Li, Moming Li, Megan Rice, Haoyuan Zhang, Dexuan Sha, Mei Li, Yanfang Su, Chaowei Yang

**Affiliations:** 1 Department of Geography and GeoInformation Science, George Mason University, Fairfax, VA 22030, USA; yli38@gmu.edu; 2 NSF Spatiotemporal Innovation Center, George Mason University, Fairfax, VA 22030, USA; 3 Department of Epidemiology and Biostatistics, University of California at San Francisco, San Francisco, CA 94158, USA; moming.li@ucsf.edu (M.L.); dsha@gmu.edu (D.S.); 4 Department of Chemistry, Carnegie Mellon University, Pittsburgh, PA 15213, USA; marice@andrew.cmu.edu; 5 Institute of Remote Sensing and GIS, Peking University, Beijing 100871, China; zhanghaoyuan@pku.edu.cn (H.Z.); mli@pku.edu.cn (M.L.); 6 Department of Global Health, Washington University, Seattle, WA 98195, USA

**Keywords:** social distancing measures, COVID-19, event study, panel data, policy analysis, mobility, mortality, spatiotemporal, heterogeneity

## Abstract

Social distancing policies have been regarded as effective in containing the rapid spread of COVID-19. However, there is a limited understanding of policy effectiveness from a spatiotemporal perspective. This study integrates geographical, demographical, and other key factors into a regression-based event study framework, to assess the effectiveness of seven major policies on human mobility and COVID-19 case growth rates, with a spatiotemporal emphasis. Our results demonstrate that stay-at-home orders, workplace closures, and public information campaigns were effective in decreasing the confirmed case growth rate. For stay-at-home orders and workplace closures, these changes were associated with significant decreases (*p* < 0.05) in mobility. Public information campaigns did not see these same mobility trends, but the growth rate still decreased significantly in all analysis periods (*p* < 0.01). Stay-at-home orders and international/national travel controls had limited mitigation effects on the death case growth rate (*p* < 0.1). The relationships between policies, mobility, and epidemiological metrics allowed us to evaluate the effectiveness of each policy and gave us insight into the spatiotemporal patterns and mechanisms by which these measures work. Our analysis will provide policymakers with better knowledge regarding the effectiveness of measures in space–time disaggregation.

## 1. Introduction

COVID-19 (SARS-CoV-2), also known as the novel coronavirus, is known to cause severe respiratory damage and other possibly fatal symptoms. COVID-19 is more fatal than the flu but has a death rate lower than other notable epidemics such as Ebola [1]. However, because coronavirus is highly contagious, it kills more people than these deadlier diseases [2]. The fact that COVID-19 is highly contagious, paired with extensive human mobility, both nationally and internationally, means that this virus has a high rate of transmission. Therefore, social distancing measures are important to implement in public areas. As the emergence and spread of this respiratory disease is aided by social contact and takes on different manifestations by region, it is important to analyze COVID-19 from a spatiotemporal perspective [3].

Social distancing relies on the basic idea that infected particles in the air are less likely to be transmitted with an increased distance between people [4]. Maintaining social distancing is important because these guidelines apply to the general population, including asymptomatic individuals who may be infectious. Unfortunately, voluntary social distancing guidelines are not sufficient to stop COVID-19 transmission. Therefore, it is imperative that governments take more concrete actions such as through mandates and closures.

To accompany social distancing recommendations, governments across the globe have enacted a series of policies including travel bans, stay-at-home orders, and quarantine periods. Many countries, for example, have issued travel bans or travel restrictions to reduce the international spread of the virus by limiting the movement of people. To prevent the spread of COVID-19 in the US, various policies have been enacted in many states, e.g., with the rapid overflow of hospital equipment and soaring number of cases, states have begun enacting “stay-at-home orders”, which, except for essential tasks or businesses, restrict the movement of residents outside of their households. These policies were likewise implemented to lower the growth rate of cases through limiting human mobility; however, they have also had unintended economic consequences. For example, almost one in four small businesses were shut down amidst the pandemic [5]. Additionally, the lockdown orders sent unemployment rates soaring to 13% by May; the highest unemployment rates since the great recession coincided with the implementation of stay-at-home orders [6].

As these policies have widely disrupted daily life, there has been much discussion surrounding the best course of action to take to combat the virus. The effectiveness of the implemented policies should be explored to examine whether they are successful in reducing the virus growth rate, and therefore whether they should remain in place for citizens to abide by, even if routine life is disrupted. Government intervention and regulation is quintessential in inhibiting the spread of COVID-19, however, many regions and urban areas in the US have implemented different policies to combat the virus, with either delayed responses or less stringent measures. This paper will examine the effects that policy implementation has on community human mobility and the growth rates of COVID-19 infection and mortality in the US.

## 2. Related Work

Many nations took different approaches to control COVID-19 as the pandemic progressed. To track variation in how quickly it took governments to react accordingly, the Oxford COVID-19 Government Response Tracker project [7] collected daily global government policies and implemented measures using a schema covering social distancing, economic, and health system policies. They also created an index to measure the stringency of social distancing measures. This policy schema became a critical guideline for many latter works. Many researchers have collected policies at fine administrative levels in various countries in accordance with this scheme, and have shared the data with the public, e.g., environmental, socioeconomic, and viral case data [8], as well as health systems, economic responses, and containment/closure data [9]. These data are valuable resources for global and regional analyses.

In addition to data collection, a series of studies have been conducted to estimate the impacts of policies on controlling the pandemic which include changes to human mobility, general characteristics of the pandemic, the mortality of the virus, and others. In the beginning of the pandemic, Wuhan was in lockdown and travel bans were quickly announced between China and other countries in an attempt to impede the nationwide and global spread of the virus. Most previous studies focused on the initial stages of the pandemic and estimated the effectiveness of policies by comparing the real virus case data after the implementation of these measures with estimated virus case data provided by simulations—such as by using a SEIR (Susceptible-Exposed-Infectious-Removed) model and its variants. Tian et al. [10] evaluated travel restrictions implemented in Wuhan City and found that travel bans were effective in slowing the spread of COVID-19 to other cities in China by 2.91 days (95% CI: 2.54-3.29). A similar study by Chinazzi et al. [11] used the global epidemic and mobility model (GLEAM) to analyze the Wuhan travel quarantine and found that this measure delayed COVID-19 progression by 3 to 5 days and contributed to a 77% decrease in cases exported from mainland China. A study by Wells et al. [12] considered travel restrictions and border control measures and estimated their impacts using a two-layer contact dispersion model. They found that in the early stages of the pandemic, international travel and border control measures were cost-effective. Similar to the previous studies, they noted that travel restriction scenarios led to an 81.3% reduction in exported COVID-19 cases over a 24-day period. 

As many other countries started to experience outbreaks, stay-at-home orders were widely adopted. These policies typically requested that citizens shelter in place for a certain amount of time, with the exception of essential errands [13]. As these orders significantly disrupted normal life, many analyses worked to estimate their effectiveness to evaluate whether their consequences were worthwhile. Using a STEx-SEIR model, researchers found that countries implementing stay-at-home orders had 30.2% less new cases after one week and a whopping 48.6% decrease after three weeks, compared to countries that did not enact these measures. These orders also contributed to a 59.8% decrease in new fatalities after a period of 3 weeks [14]. 

Although social distancing is the principle guideline for reducing the transmission speed of COVID-19, governments have issued different variations of social distancing measures depending on the COVID-19 situation and culture, when nationwide quarantine policies are not possible. To help decision makers evaluate the impact of polices in a certain area, some research has focused on specific geographic and political jurisdictions. For example, Gupta et al. [15] focused on the policies created in state and local areas in the US while using cellular data to track human movement and found that all regions showed a large reduction in mobility. Furthermore, they noted that policies implemented earlier resulted in the greatest mobility reductions. Overall, they concluded that the decrease in mobility was a result of both governmental interventions and individual motivations to remain distant. Badr et al. [16] used mobility data to generate a social distancing metric. Specifically, they analyzed how changes in mobility affected the virus’ growth rate in 25 US counties. Their study found a strong correlation between social distancing and decreases in the growth rate of the virus and therefore concluded that social distancing was an effective measure. 

In contrast to the previously mentioned research, which focused on analyzing the impacts of specific measures, Courtemanche et al. [17] focused on determining which social distancing policies were the most effective in reducing the spread of the virus among four governmental interventions including shelter-in-place orders, public school closures, bans on large social gatherings, and closures of entertainment-related businesses. They discovered that shelter-in-place orders and the closure of public places slowed COVID-19 spread the most. Interestingly, this research found that school closures and bans on large gatherings did not show a significant effect. This study did not include other potentially important policies such as workplace closures and public information campaigns and only used short-term data from March to April. Additionally, this study only focused on the impact of policies in regard to confirmed cases, which might be underestimated due to the lack of diagnostic capacity in March and April. Our study will include a wider analysis period and will also investigate mortality as it is another insightful epidemiological metric. Since actual confirmed and death cases are higher than reported values [18], studying different policies is insightful as their effects may help control both the reported and undetermined actual values.

In our study, three conceptual mobility subgroups, potentially representing different levels of policy effects, are defined and incorporated. Furthermore, we expand upon previous studies by introducing additional policies used in the US according to the schema defined by the Oxford COVID-19 Government Response Tracker project [7]. These measures—school closures, workplace closures, public event cancellations, public information campaigns, public transport closures, stay-at-home orders, and international/national travel controls—represent more concrete and enforceable types of social distancing measures.

Using this set of policies will help us achieve the objective of our study—characterizing a more holistic picture of the effectiveness of different types of policies in reducing human mobility and the COVID-19 confirmed and death case growth rates. Previous studies proposed intriguing relationships between COVID-19, policy, and mobility. Some analyzed policies in respect to transmission metrics while others looked at how policies affected mobility. However, previous studies did not comprehensively explore all of these concepts. This analysis will help us understand the current spatiotemporal connections between policies, mobility, and COVID-19 trends in the US. Specifically, state-level data are used since most counties follow policies implemented by the state government. This research aims to provide a thorough, spatiotemporal, and quantitative understanding of social distancing policies and their impacts, which can support policy making regarding COVID-19 and other infectious diseases in the future.

## 3. Data

### 3.1. State-Level Policy Data

Our analysis utilized state-level policies to examine the effectiveness of measures used to combat COVID-19 in the US. The policy data were shared by the NSF spatiotemporal center [8], which adopted the schema defined by the Oxford COVID-19 Government Response Tracker project [7], containing seven designated categories, as seen in Table 1. If a specific policy issued by a governmental agency did not fall into one of these categories, it was not accounted for in this study. The whole dataset, which was obtained from Github (https://github.com/stccenter/COVID-19-Data/tree/master/Policy/US_Policy), indicates the extent of the US government’s responsiveness to the outbreak of COVID-19 and the status of policies in specific areas throughout the lockdown. Our study used policies from March 1 to July 13.

### 3.2. Mobility Data

Google state-level community mobility reports were utilized in this study to analyze policy impacts. Mobility data were chosen as they represented behavioral changes that were not associated with pharmaceutical interventions, but merely policy implementation [20]. The mobility reports included dynamic changes in visits to different areas. To compute the change in mobility, daily movements during the pandemicof COVID-19 were compared to human’s normal movements in a baseline period—between 3 January and 6 February 2020. The percentage difference of movement between the date of interest and the baseline was calculated from Google map usage data.

For each state, Google community mobility data (https://www.google.com/covid19/mobility) contained changes in six mobility categories: grocery and pharmacy, parks, transit stations, retail and recreation, residential, and workplaces. These groups allowed researchers to understand changes in mobility resulting from different policies. For example, grocery and pharmacy were combined as both were deemed essential outings. Trends in each category for a specific location and time were reported. Most mobility data referred to the number of visits to each location. However, residential data included a comparison of the length of stay at various places.

### 3.3. State-Level Confirmed Case and Death Data

State-level COVID-19 confirmed and death case data were also obtained from the National Science Foundation (NSF) Spatiotemporal center Github repository [21] (https://github.com/stccenter/COVID-19-Data/tree/master/US). Both the cumulative confirmed and death cases were listed daily. Because our study aims to estimate the impact of policies, growth rates for both confirmed and death cases were derived from the original dataset and used in the subsequent analysis.

### 3.4. Control Variables

Additional control variables related to COVID-19 were added in this study, including daily counts of positive and negative tests in each state, which were added together to represent the total number of people tested for COVID-19 every day. This parameter was taken into consideration because the number of confirmed cases is restricted by the testing capability of each state. Daily pending data were not included in this study. Time-invariant factors, such as the population density, number of ICU beds, size of medical staff, etc., were not directly included in our model (detailed in the statistical analysis subsection). State-level population totals, however, were integrated into our analysis (through regressions) to weight the observations to reflect the individual demographic differences of each state.

## 4. Methods

There are two main features of the collected data. First, data were collected for different states over time, resulting in a panel data structure [22,23]. Secondly, several policies were initiated at different dates and had different durations. More importantly, policies have delayed/duration effects in the confirmed/death case growth rates. To properly model and examine the policy effects, we utilized the regression-based event study technique [24]. Event studies have been widely applied in economics, finance, and other social science disciplines [17,25,26,27,28], and have demonstrated to be a powerful tool for policy analysis. In this analysis, we apply the event study method to estimate what the normal mobility and mortality should be at the day of the events as well as several days prior and after the events (i.e., during the event window). Thereafter, this method deducts this “normal” from the “actual” to receive the “disrupted mobility/mortality” attributed to the event.

**Figure 1:**
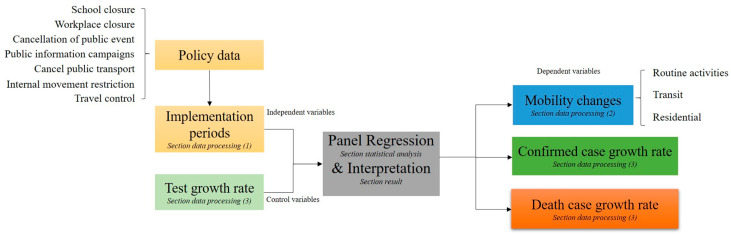
The workflow of the policy analysis.

While presenting the regression specification in the statistical analysis subsection, we outline the general framework of our quantitative analysis here. Figure 1 shows the workflow consisting of four main steps:

- collecting policy, mobility, and case data, and conducting respective transformations (data processing subsection);
- conducting regression analysis on mobility changes, confirmed case growth rates, and death case growth rates (statistical analysis subsection);
- analyzing spatiotemporal trajectories across the US (Appendix A);
- estimating the impact of policy measures and interpreting the results. In addition, state-specific time trends are analyzed.

### 4.1. Data Processing

#### 4.1.1. Policy

A state-level policy stringency index that measures the responsiveness of each region’s governmental COVID-19 response was computed based on collected policy data according to the definition in the Government Response Tracker project [7]. To simplify the model, policy was encoded as a 0–1 variable, indicating whether the policy was active or not on day d. For the encoded 0–1 policy variables, a duration indicator *i*, ranging from -1 to 6 (normalized to 0, the reference level), was generated to indicate the time range of the policy as shown below (1), where *t* counted the cumulative days prior to day *d* since the policy’s initiation.

(1) $$i=\;\{\begin{array}{c} -1,\;if\;t\leq -8\;\\ 0,\;if\;t\geq -7\;and\;t\leq -1\;\\ 1,\;if\;t\geq \;1\;and\;t\leq \;7\;\\ 2,\;if\;t\geq 8\;and\;t\leq 14\\ 3,\;if\;t\geq 15\;and\;t\leq 21\\ 4,\;if\;t\geq 22\;and\;t\leq 28\\ 5,\;if\;t\geq 29\;and\;t\leq 59\\ 6,\;if\;t\geq 60\;\; \end{array}$$

#### 4.1.2. Mobility Report

Google’s COVID-19 Community Mobility data were divided into three subgroups, motivated by previous studies [20,29] and their underlying assumptions (https://mrc-ide.github.io/covid19usa). Subgroup 1, named routine activities, is an average of retail and recreation, grocery and pharmacy, and workplace categories, and reflects their effects on mobility at both countrywide and region-specific levels. Subgroup 2, consisting of transit reports, determines both countrywide and state-specific effects of mobility restrictions. Whereas subgroup 3, residential reports, represents a nationwide effect of policy on mobility. These studies relied on the basis that these three subgroups played critical roles at different spatial levels when used to model COVID-19 confirmed cases and death cases. Our analysis, on the other hand, demonstrates that these subgroups may provide us with insight into the specific spatial level of policy effects pertaining to the mobility-influenced COVID-19 confirmed and death case growth rates.

#### 4.1.3. Confirmed/Death Case Report

To measure the impacts of state-level policies, confirmed and death case growth rates were calculated using the cumulative confirmed and death cases, respectively. First, daily cumulative case counts that were below a threshold (100 in this study) were discarded for each state. These initial counts represented the so-called spark risk, which is the risk associated with the emergence of a pandemic; our analysis was instead concerned with the spread risk—the likelihood of a pandemic to diffuse [30]. Spark risk typically provokes governments to adopt medical and policy interventions. On the other hand, the effectiveness of these policies is exhibited during the spread risk stage. To obtain the growth rate on a specific day, the difference between the logarithms of the cumulative counts for this day and the prior day was calculated, as this is a widely used approximation for the growth rate. The growth rate was then multiplied by 100, enabling the estimated regression coefficients for the policy duration indicators to be interpretated as percentage point changes. The definition of the death case growth rate is similar to that for the confirmed cases (2).

(2) $$ConfirmedGR_{sd}=\;\ln\left(confirm_{sd}\right)-\ln\left(confirm_{s,d-1}\right)$$

### 4.2. Statistical Analysis

**Figure 2:**
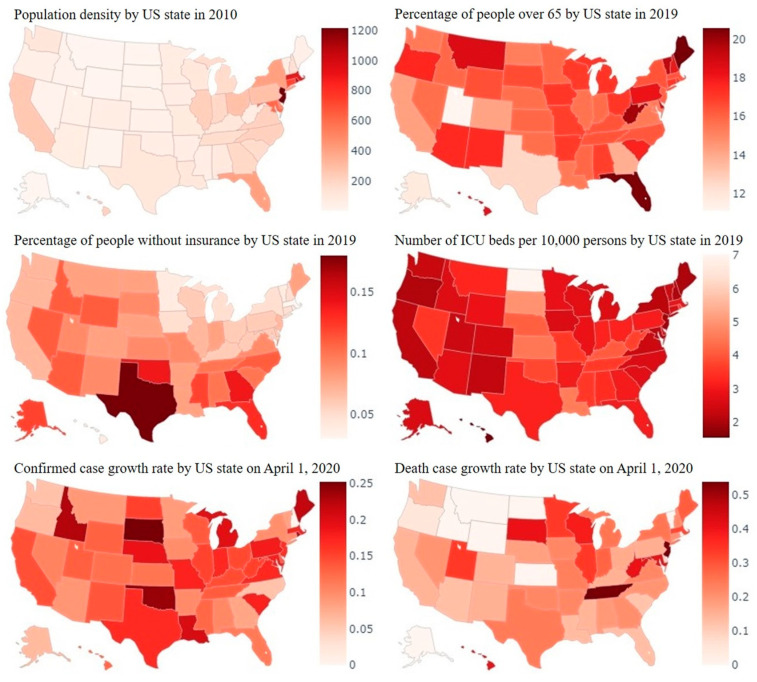
Some representative factors related to the severity of COVID-19 and their heterogeneous paths.

In the current study, we focus on the fixed effects panel regression model, which is advantageous in that unobserved heterogeneity can be modeled through state-specific fixed effects. As shown in Figure 2, certain factors are heterogeneous among states, such as the old-age population distribution and medical capacity. Variation and association can be seen when these factors are related to the confirmed and death case growth rates, e.g., states with less of their population over 65 and states with more ICU beds per 10,000 people generally have smaller death case growth rates.

The response variables in this analysis were human mobility and confirmed and death case growth rates. From exploratory analysis, we saw a certain declining pattern in the growth rates and an increasing pattern in mobility over time—although the actual patterns may vary across states. In addition to the state fixed effects, this motivated us to control for the possible effects of time in the panel regression model. Therefore, we incorporated state-specific trends, which captured individual states’ tendencies over time. Exploratory analysis showed that mobility changes over time roughly followed a linear pattern, while the confirmed and death case growth rates had non-linear features. A particular non-linear trend was chosen (3,4) over other functional forms, as it produced better fits to the data and provided more interpretable results. A further discussion of the state-specific time trends is presented in the results section. 

The seven policies in the main study of interest were denoted by *school, work, event, transport, campaign, home, and travel*. We first assumed the model for the confirmed case growth rate is as follows (3).

(3) $$\begin{array}{ccc} ConfirmedGR_{sd} & = & \alpha +state_{s}\\ & + & (\underset{i\neq 0}{\sum }\beta_{1i}school_{sdi}+\underset{i\neq 0}{\sum }\beta_{2i}work_{sdi}+\underset{i\neq 0}{\sum }\beta_{3i}event_{sdi}+\underset{i\neq 0}{\sum }\beta_{4i}transport_{sdi}\\ & + & \underset{i\neq 0}{\sum }\beta_{5i}campaign_{sdi}+\underset{i\neq 0}{\sum }\beta_{6i}home_{sdi}+\underset{i\neq 0}{\sum }\beta_{7i}travel_{sdi})+\gamma TestGR_{sd}+\zeta_{s}me^{-m}\\ & + & \epsilon_{sd} \end{array}$$

In the above equation, $state_{s}$ denotes the unobserved fixed effect for state *s*, $school_{sdi}$ is the indicator variable for the policy *school* belonging to the period *i* for state *s* and day *d* (the remaining 6 policies are defined similarly), $TestGR_{sd}=\ln\left(test_{sd}\right)-\ln\left(test_{s,d-1}\right)$ is the test growth rate for state *s* and day *d*, $\zeta_{s}me^{-m}$ denotes the state-specific monthly non-linear time trends (state *s* and month *m*), and $\epsilon_{sd}$ is the additive error for this linear regression model. To account for a possible clustering effect, where data were collected repeatedly over time on a state-level basis, we adjusted the observations with the state population and robust standard errors clustered by states were estimated. The models for the death case growth rate and mobility change are presented below (4) and (5), respectively, where *k* indexes the three mobility subgroups. In addition to the test growth rate, we included the confirmed case growth rate as a control variable for the death case growth rate response variable (4).

(4) $$\begin{array}{ccc} DeathGR_{sd} & = & \alpha +state_{s}\\ & + & (\underset{i\neq 0}{\sum }\beta_{1i}school_{sdi}+\underset{i\neq 0}{\sum }\beta_{2i}work_{sdi}+\;\;\;\underset{i\neq 0}{\sum }\beta_{3i}event_{sdi}+\underset{i\neq 0}{\sum }\beta_{4i}transport_{sdi}\\ & + & \underset{i\neq 0}{\sum }\beta_{5i}campaign_{sdi}+\underset{i\neq 0}{\sum }\beta_{6i}home_{sdi}+\;\;\;\underset{i\neq 0}{\sum }\beta_{7i}travel_{sdi})+\gamma TestGR_{sd}\\ & + & \eta ConfirmedGR_{sd}+\zeta_{s}me^{-m}\;+\epsilon_{sd} \end{array}$$

(5) $$\begin{array}{cc} Mobility_{sd}^{k} & =\alpha +state_{s}\\ & +(\underset{i\neq 0}{\sum }\beta_{1i}school_{sdi}+\underset{i\neq 0}{\sum }\beta_{2i}work_{sdi}+\underset{i\neq 0}{\sum }\beta_{3i}event_{sdi}+\underset{i\neq 0}{\sum }\beta_{4i}transport_{sdi}\\ & +\underset{i\neq 0}{\sum }\beta_{5i}campaign_{sdi}+\underset{i\neq 0}{\sum }\beta_{6i}home_{sdi}+\underset{i\neq 0}{\sum }\beta_{7i}travel_{sdi})+\zeta_{s}m\;+\epsilon_{sd} \end{array}$$

## 5. Results

### 5.1. Impacts of Policies on Mobility, COVID-19 Cases, and Mortality

Stay-at-home orders were associated with significant decreases in mobility in the first three weeks. For example, after the implementation of stay-at-home orders, the amount of time people stayed in residential areas increased by 1.088 percentage points in the first week, 1.255 in the second week, and 1.697 in the third week. The impacts of this policy on mobility were insignificant by the fourth week, however, the impacts of stay-at-home orders on the confirmed case growth rate were significant over four months. Specifically, these impacts showed a percentage point reduction of 4.787 by the fourth week. Impacts on the death case growth rate were not significant in the first four weeks but became significant afterwards, with a reduction of 5.065 percentage points in the second month and a reduction of 6.533 percentage points after two months. 

Significant decreases (*p* < 0.05) in mobility were also observed with workplace closures within the first month (Table 2). Similar to the stay-at-home orders, workplace closures also resulted in significant decreases (*p* < 0.05) in the confirmed case growth rate, however, these results represented a longer horizon (e.g., a reduction of 6.324 percentage points in the third week and a reduction of 7.350 percentage points over two months). The death case growth rate for workplace closures was not significant in any analysis interval. Again, changes in mobility were not persistent, however, these short-term impacts may limit the ability of these types of policies to create dramatic, long-lasting, mobility-induced impacts regarding the case growth rates.

Public event cancellations also saw significant (*p* < 0.05) changes in mobility in all subgroups for the first few weeks (e.g., reductions of 5.602, 6.489, and 6.674 percentage points in transit group in weeks one, two, and three, respectively). This policy saw significant results in the confirmed case growth rate after one month—there was a decrease of 6.681 percentage points between one and two months (*p* < 0.1) and a decrease of 7.281 percentage points over two months (*p* < 0.05). Our analysis could not find evidence that public event cancellations had significant impacts on the death case growth rate.

Similar mobility patterns were observed for policies regarding public transport closures. In fact, among all polices considered, public transport closures resulted in the largest percentage changes in all mobility subgroups (*p* < 0.01). As their impacts on mobility changes were especially significant, public transport policies could effectively and rapidly reduce human mobility at state, regional, and national levels. However, no significant impacts were observed for this policy in regard to either case growth rate which suggests that mobility may not always directly affect COVID-19 trends. 

On the other hand, the impacts of school closures and international/national travel controls on mobility were not significant. Likewise, they had only limited impacts on each case growth rate. Overall, most policies showed a decrease in the death case growth rate, except for school closures, but their coefficient estimates were not statistically significant. Although a clear majority of these policies were effective in controlling the confirmed case growth rate, they were less promising in reducing the death case growth rate. Indeed, death cases are affected by many other non-policy factors, such as medical resources, the distribution of age/race groups, etc.

Our analysis showed that public information campaigns generally had little to no effect on the mobility subgroups; however, these measures can effectively reduce the COVID-19 confirmed case growth rate (e.g., 10.331 percentage points in one week, 18.792 in two weeks, 19.538 in three weeks, 18.569 in four weeks, 19.917 in one to two months, and 22.534 in more than two months). Not only were these changes the most significant (*p* < 0.01) out of any policy category, but they were also the largest. This shows that policy can significantly impact COVID-19 trends through channels other than changes in mobility. Similar mechanisms may play a role in stay-at-home orders as both of these policy types can be considered health system policies. Contrary to medical system policies, health system policies primarily affect people’s behavior and limit their mobility by increasing their awareness and changing their perceptions of the pandemic. For example, people will be more likely to wash their hands and wear a mask if health system policies promote protective strategies individuals can take—ultimately reducing COVID-19 transmission. 

It was also interesting to note that the effectiveness of social distancing policies generally diminishes overtime. Many of the policy categories saw their most significant mobility changes within the first few weeks. Compliance with policies may be reduced over time as people become restless. Indeed, many of the policies that exhibited this trend (e.g., stay-at-home orders, workplace closures, and public event cancellations) showed decreasing or limited significance in their case growth rates overtime. This again emphasizes the critical role that health system measures, such as public information campaigns, play in mitigating COVID-19 through non-mobility related mechanisms. Furthermore, since the effects of social distancing measures on mobility diminish overtime and in turn, they have less of an impact on case growth rates, it is vital to continually enforce many types of mitigation mechanisms that are not limited to mobility, such as public information campaigns.

### 5.2. State-Specific Time Trends

**Figure 3:**
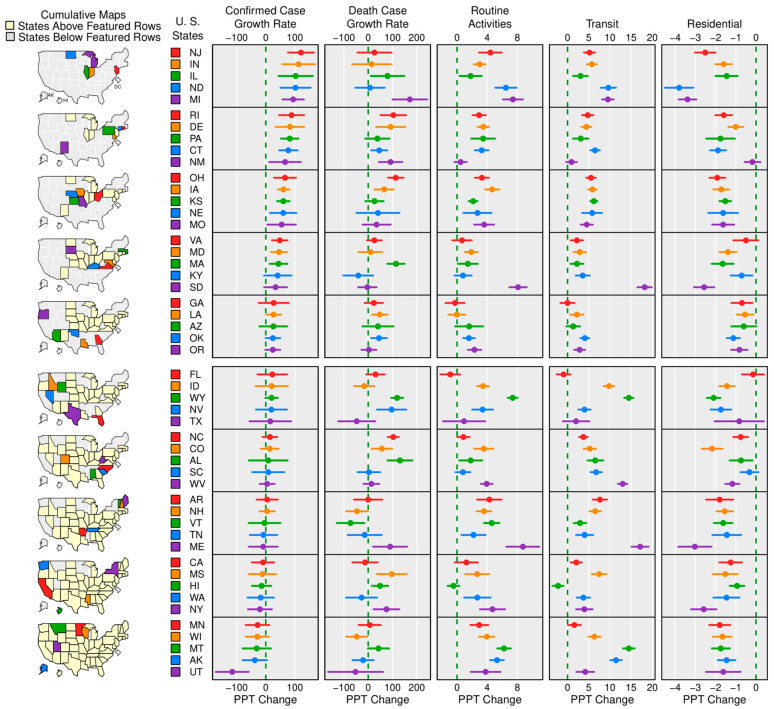
Counterfactual state-specific time trends in absence of seven social distancing policies.

Figure 3 shows the counterfactual state-specific time trend in regard to changes in the three mobility subgroups, as well as the confirmed and death case growth rates, after controlling for policies and non-policy effects. Specifically, the five columns display the estimated coefficients (with 95% confidence intervals) for the time trends modeled in the aforementioned equations. Since the time trend for the confirmed and death case growth rates is a non-linear decreasing function during the study period (March to July), a positive estimated coefficient would indicate a decrease in the growth rate, while a negative estimated coefficient would indicate an increase. On the other hand, since we used a linear trend for the three mobility subgroups, a positive estimated coefficient would indicate that the growth rate increases over time. Spatial heterogeneity can easily be seen via diverse patterns between these state-specific trends. Significant positive trends can be seen for most states in the routine and transit mobility subgroups. While oppositely, these results coincided with those for the residential mobility subgroup where most states showed a significant negative trend. 

**Figure 4:**
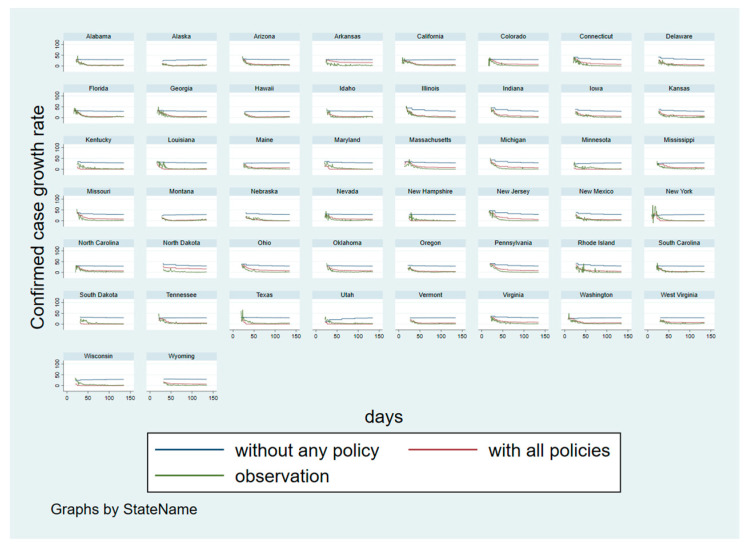
Counterfactual analysis: state-specific time trends for the confirmed case growth rate with and without social distancing measures.

Careful readers may find that some of the results in Figure 3 are not representative of reality—some states that have been considered to be performing well in mitigating COVID-19 may display opposite results in our analysis. For example, Hawaii has one of the lowest infection rates from COVID-19 and has some of the strictest quarantine rules out of all states [31]. In contrast, our analysis showed that Hawaii had an increasing confirmed case growth rate trend. On the other hand, our data analysis indicated that New Jersey’s confirmed case growth rate decreased the fastest. In reality, New Jersey has the highest population density with a relatively large number of infections. In some instances, decreased mobility in the routine and transit subgroups may be associated with increasing case growth rate trends as well. Figure 3 helps explain these seemingly contradictory results as it displays the time trends after controlling for policy and other non-policy effects. In other words, policy effects are held unchanged in this figure. We further demonstrate this point in Figure 4, which plots the fitted regression curves with all policies against their observed counterparts. This figure shows that our models are reasonable and capable of producing satisfactory results. Most states—with the exception of Arkansas and North Dakota—show good fits between the predicted confirmed case growth rate that considers all policies and the actual observations. As a byproduct, we can also investigate the hypothetical situation where there is no policy via the counterfactual analysis. One apparent feature displayed for most states in Figure 4, is the large gap between the predicted curves with and without policies. This unequivocally reveals that social distancing policies have a substantial impact in reducing confirmed case growth rates. Additionally, there is a wide heterogeneity among these curves which further emphasizes that it is necessary and reasonable to consider state-specific time trends to model diverse time effects.

**Figure 5:**
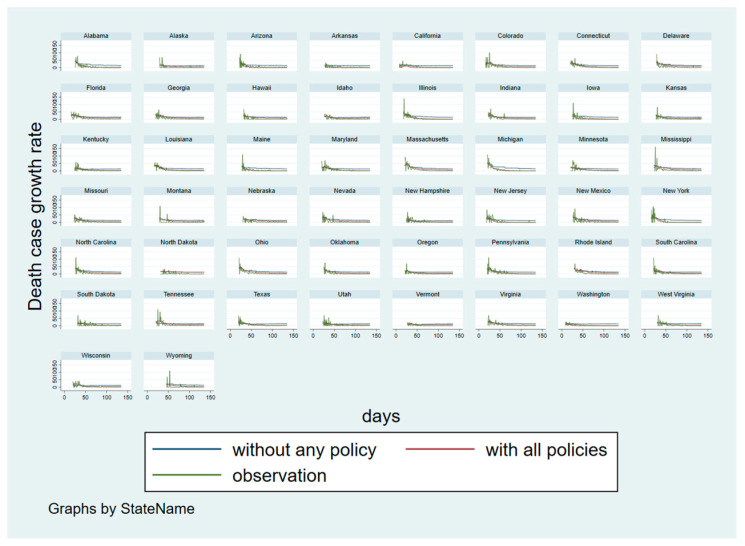
Counterfactual analysis: state-specific time trends for the death case growth rate with and without social distancing measures.

Figure 5 displays the same state-specific time trends but in respect to the death case growth rates. Like Figure 4, the fits of the predicted curves with the real observations are satisfactory—even though large fluctuations are seen in the beginning of the analysis period in some states. However, the gap between the predicted curves with and without policy is narrower than the gap seen in Figure 4. This supports our findings and assertion that policies have less of an impact on the death case growth rate than the confirmed case growth rate.

Figure 4 and Figure 5 are also represented in Appendix C with a 7-day moving average to smooth the raw observations. Even with smoothing, the results are not substantially different from what is observed here. Furthermore, discrepancies between observed and fitted values still remain for the states of Arkansas and North Dakota when using smoothed data, which suggests that the poor predictions for certain states cannot be completely attributed to fluctuations in the unsmoothed data. Even though our model is limited in that a few states are predicted poorly, its performance is satisfactory in most cases. Therefore, the model we have established could be used for further investigations such as to assess how the growth rate may change if only certain policies are implemented at a time.

## 6. Discussion

This study estimated the impacts of seven social distancing policies on changes in human mobility and the growth rates of confirmed and death cases in the US. These results can help emphasize the parts of policies which are the most fundamental in combatting COVID-19. Results show that policies which create incentives for less movement and prohibit large gatherings—such as stay-at-home orders, workplace closures, public event cancellations, and public transport closures—decrease mobility the most significantly (*p* < 0.05 in most duration periods). This reveals that removing factors that drive people to enter public venues (e.g., public event cancellations) would lead to a great reduction in mobility. With public transport closed, people may have a harder time moving to places other than their current residences which inhibits mobility. Likewise, with workplaces closed, people are likely to work and spend more time in residential areas. Furthermore, stay-at-home orders discourage people from moving outside their households which may reinforce the mobility changes seen with other policies, such as by limiting the need for public transportation. Interestingly, after the implementation of social distancing policies for several months, the mobility of routine activities and transit increased, while the time spent in residential areas decreased, although people still move less than they did before the pandemic. This result demonstrates that governments should continue to remain stringent on policies and increase public awareness regarding mobility-induced transmission.

In regard to the COVID-19 confirmed case growth rate, stay-at-home orders and workplace closures led to significant decreases (*p* < 0.05) in the first few weeks. This supports the assertion described in previous studies, that policies which limit mobility can in turn control disease transmission [16]. Although public information campaigns did not significantly affect mobility, they did produce significant decreases (*p* < 0.01) in the confirmed case growth rate. This emphasizes the importance of implementing health system policies that may not influence COVID-19 trends directly through mobility. These types of policies can influence a population’s knowledge and willingness to comply with health measures that aim to limit COVID-19 transmission. Indeed, many media outlets have agreed that a coordinated “public information campaign that reinforces key messages to shape people’s behaviors and prevent the spread of the virus” is needed given the current progress of the pandemic [32]. Increased knowledge has been seen to have a positive impact on risk perception [33]; if people are more aware of COVID-19 risk they may be more likely to follow mitigation measures. Additionally, sources [34] have noted that social media and news sources are essential in helping spread this information to a large audience. Indeed, communication between policy makers and the public represents another class of effective mechanisms that should be utilized in mitigation efforts [35].

Policies regarding school closures and international/national travel controls resulted in decreases in most of the variables examined. However, most of their impacts were statistically insignificant. A study on control measures taken during the Beijing SARS outbreak in 2003 noted that school closures were not very effective in controlling disease transmission [36]. Two other studies [17,37] could not find significant decreases in the growth rates resulting from school closures, either. Another study similarly reported that travel restrictions were effective in the early stages of the pandemic but became ineffective for most countries/regions as the epidemic progressed [38].

The results of this study also highlight the intrinsic spatiotemporal heterogeneity in the US. For example, the impacts of stay-at-home orders on mobility diminished over time while a one-month lag was observed in regard to the death case growth rate impacts. Indeed, the duration and extent of significance seen in these policy-outcome relations provides us with insight into the connections between policies and the mechanisms by which they affect COVID-19 trends.

Noticeably, these results should be deciphered under the assumption that many infected individuals were not accounted for; testing was not at full capacity and asymptomatic individuals are less likely to get tested. Therefore, the growth rates used may be larger than estimated. Additionally, our study converted all seven policies to 0–1variables to simplify the modeling and data analysis. Additional stringency levels, which could be defined by whether a policy is recommended or required, could greatly increase the total amount of policy variables (e.g., the generated policy duration indicators). This may make the model structure redundant and its interpretation opaque. It is therefore convenient to consider combined indices of several policies, where multiple categorical policy variables are re-weighted to form a continuous index used for further analysis. Similarly, other types of measures (e.g., mask usage, behavioral measures, mandatory testing, mandatory quarantines) could have been included to represent a broader range of COVID-19 mitigation strategies [39,40]. Furthermore, future research could use alternative data to measure policy (e.g., Oxford policy data) and mobility (e.g., Descartes, Facebook mobility, SafeGraph) to validate and expand upon our findings. 

This study also focused on the short-term impacts of policies. For example, analyzing COVID-19 confirmed cases in July 2020 raises the possibility that short-horizon, time-contingent, and state-contingent policies can effectively and temporarily reduce mobility and mortality. Long-horizon policies warrant future research as they might mute the mobility and mortality responsiveness due to changes in the perception of COVID-19, spillover across states, and other factors. Lastly, it is worth mentioning that several existing studies, including this current one, could not detect significant policy effects regarding school closures. This motivates us to consider model-based approaches such as the SEIR model or other agent-based models to study potential policy effects that have not yet been discovered. These models may be useful in determining the best ways for schools to reopen (e.g., some schools in Georgia and North Carolina have attempted to reopen but have been unsuccessful) and the potential consequences that may result from the easing of policies.

## Figures and Tables

**Table 1:** State-level policy data in the US.

Policy	Description	Stringency
School closure	A report of the closing of schools and universities beginning in Ohio on 12 March 2020 [19]	0—no measurement 1—recommended 2—required
Workplace closure	A report of the closing of places of work. 19 March 2020 was the beginning of workplace closures in the US [7]	0—no measurement 1—recommended 2—required
Public event cancellation	A report of the cancellation of public events in a state. By 6 March 2020 some music festivals had been canceled [19]	0—no measurement 1—recommended 2—required
Public transport closure	A report of the closing of public transportation. Transport closures were recommended by 17 March 2020 [7]	0—no measurement 1—recommended 2—required
Public information campaign	Implementations of state-level public information campaigns regarding COVID-19. Public information campaigns started to be put in place by 16 March 2020 [7]	0—no campaign 1—campaign held
Stay-at-home order	Stay-at-home orders were collected for the category of internal movement restrictions. The 15 March 2020 marked the beginning of stay-at-home orders for non-essential workers in the US [7]	0—no measurement 1—recommended 2—required
International/national travel control	A record of international/national travel controls. On 30 January 2020 the US started implementing COVID-19 related travel advisories [19]	0—no measure 1—screening 2—quarantine on high risk regions 3—ban on high risk regions

**Table 2:** The impact of policy on human mobility, COVID-19 cases, and mortality of COVID-19.

	Routine Activities (5)	Transit (5)	Residential (5)	Confirmed Case Growth Rate (3)	Death Case Growth Rate (4)
Stay-At-Home Order					
1 week	−2.915 **	−2.801 **	1.088 **	−5.000 ***	0.714
2 weeks	−2.286	−3.737 **	1.255 **	−5.461 ***	−2.495
3 weeks	−3.780 **	−4.333 **	1.697 ***	−5.202 **	−3.751
4 weeks	−0.957	−1.252	0.866	−4.787 **	−4.396
One to two months	0.538	−0.003	0.423	−3.731 *	−5.065 *
More than 2 months	3.436	2.677	−0.555	−3.737 *	−6.533 **
Workplace Closure					
1 week	−4.201 **	−4.565 **	1.604 **	−1.497	0.928
2 weeks	−5.172 **	−5.284 **	2.157 **	−4.577 *	−3.236
3 weeks	−5.833 **	−5.882 **	2.729 **	−6.324 **	−1.941
4 weeks	−7.776 ***	−6.953 **	3.473 ***	−7.524 ***	−2.048
One to two months	−6.773 **	−5.718 *	3.542 ***	−7.414 **	−1.603
More than 2 months	−4.748	−3.934	2.171	−7.350 **	−1.676
School Closure					
1 week	1.425	0.397	−0.087	3.711	2.201
2 weeks	−0.812	0.018	0.282	3.935	6.005
3 weeks	0.092	−0.094	−0.277	3.186	6.123
4 weeks	0.250	−0.648	−0.638	2.970	6.680
One to two months	0.951	0.182	−1.239	2.142	6.414
More than 2 months	2.693	2.599	−1.986	2.103	5.981
Public Event Cancellation					
1 week	−5.602 ***	−4.689 **	2.160 ***	2.999	3.588
2 weeks	−6.489 **	−7.076 ***	2.987 ***	−0.071	1.646
3 weeks	−6.674 **	−8.421 ***	3.138 ***	−2.483	−2.034
4 weeks	−5.809	−8.303 **	2.877 **	−4.263	−3.919
One to two months	−3.684	−7.021 *	2.199	−6.681 *	−6.169
More than 2 months	−2.831	−4.444	1.719	−7.281 **	−6.24
Public Transport Closure					
1 week	−10.470 ***	−8.884 ***	3.256 ***	−2.811	−2.029
2 weeks	−12.559 ***	−10.242 ***	3.846 ***	−1.326	0.129
3 weeks	−12.433 ***	−10.975 ***	4.690 ***	−1.609	−0.585
4 weeks	−17.036 ***	−14.509 ***	5.555 ***	−3.147	−1.62
One to two months	−16.150 ***	−14.477 ***	5.315 ***	−3.595	−2.284
More than 2 months	−17.015 ***	−15.207 ***	5.285 ***	−3.304	−1.313
International/National Travel Control					
1 week	0.889	0.489	−0.295	−2.834	−7.746 *
2 weeks	0.483	−0.905	−0.188	−4.227	−6.293
3 weeks	0.487	−1.353	−0.367	−4.375	−7.351
4 weeks	1.439	−0.583	−0.423	−3.784	−7.107
One to two months	2.905	0.388	−1.131	−3.795	−8.047
More than 2 months	3.920	2.508	−1.586	−2.927	−8.596
Public Information Campaign					
1 week	−2.847	−1.379	0.884	−10.331 ***	2.661
2 weeks	−6.576 *	−4.196	2.109	−18.792 ***	0.572
3 weeks	−7.467 *	−5.834	2.078	−19.538 ***	−0.426
4 weeks	−8.410 *	−6.031	2.248	−18.569 ***	1.112
One to two months	−7.375	−5.281	1.791	−19.917 ***	−1.653
More than 2 months	−6.176	−4.195	1.511	−22.534 ***	−2.202
Test growth rate				−1.096	3.344
Confirmed growth rate					32.422
constant	−38.893 ***	−60.019 ***	23.337 ***	28.984 ***	12.484
r2	0.781	0.810	0.506	0.792	0.518

Notes: * *p* < 0.1, ** *p* < 0.05, *** *p* < 0.01; State-specific time trends corresponding to this table are shown in Table A1.

**Figure A1:**
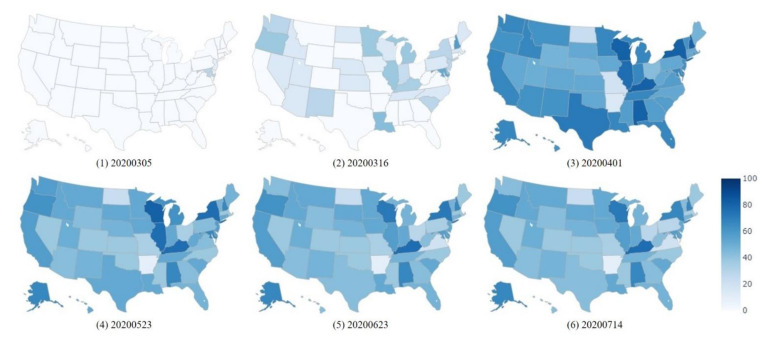
Policy stringency in the US during the epidemic of COVID-19.

**Figure A2:**
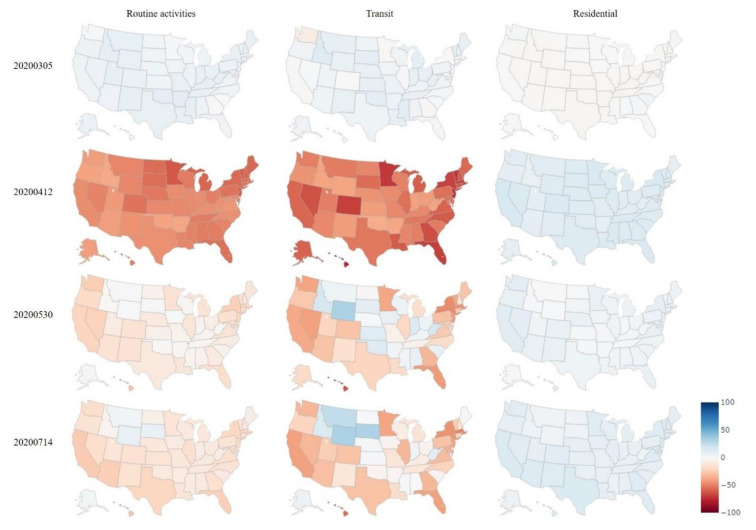
Community mobility changes during the epidemic of COVID-19 across the US.

**Figure A3:**
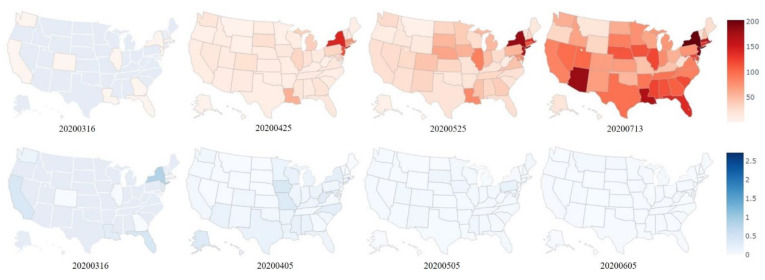
Confirmed COVID-19 cases per 10,000 people (top) and growth rate (bottom) across the US.

**Figure A4:**
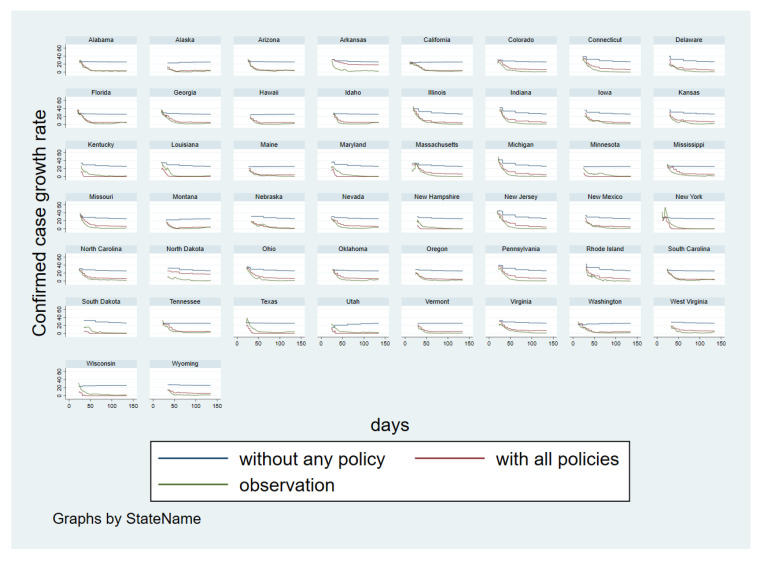
Counterfactual analysis: state-specific time trends for the confirmed case growth rate with and without social distancing measures based on smoothed data (7-day moving average).

**Figure A5:**
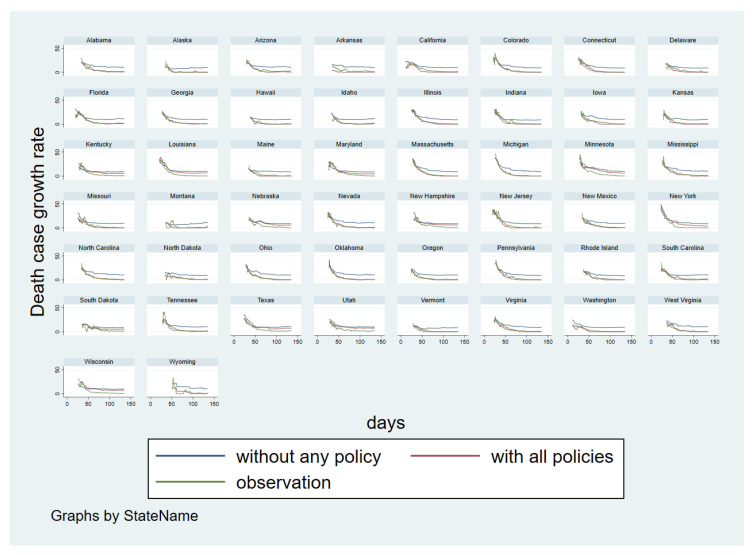
Counterfactual analysis: state-specific time trends for the death case growth rate with and without social distancing measures (7-day moving average).

## Data Availability

https://github.com/stccenter/COVID-19-Data.

## Appendix A. Descriptive Spatiotemporal Trajectory

The map below displays the stringency index in each US state at different points in time. This index serves as a valid scope in detailing the response level of state governments as they worked to mitigate COVID-19. On March 5, Maryland declared a state emergency (Figure A1(1)) [8], implemented COVID-19 public information campaigns, and closed K-12 schools. As the virus spread across the US, other states took similar actions. By 1 April, most states had implemented stay-at-home orders, cancelled large public gatherings, and closed nonessential businesses. These actions aimed to slow the spread of the virus by keeping physical distances between people (Figure A1(2,3)). Despite increasing cases throughout the spring, starting in May, several states decreased their policy stringency due to economic concerns (Figure A1(4–6)). Big spatiotemporal data were essential for our analysis in order to highlight trends across the US over time [41].

Each of the mobility subgroups had a different net change in their relative movement. As a result of regulations prohibiting unnecessary public movement, there was a notable increase in time spent in residential areas. All other mobility categories in most states saw a significant decrease in their movement, especially in April 2020 (Figure A2). The largest negative percent changes were for the transit and routine activity groups. Interestingly, there was a slight rise in routine activities and transit mobility after several weeks corresponding to the easing of restrictions.

The maps in the top row of Figure A3 depict confirmed cases of COVID-19 per 10,000 people in the US on different days, between March 2020 and July 2020. At one point, New York was deemed an epicenter of COVID-19 spread in the US; its growth rate of confirmed cases was the largest in March and April and has since been reduced. Similarly, the growth rate decreased in most states during the analysis timeframe. Due to limited space in this paper, the death case spreading maps and growth rate maps are not included.

## Appendix B. Regression Estimates for State-Specific Time Trends Based on Raw Observations

**Table A1:** State-specific time trends (as part of Table 2 in the main text).

Time Trend	Routine Activities (5)	Transit (5)	Residential (5)	Confirmed Case Growth Rate (3)	Death Case Growth Rate (4)
Alabama	1.818 **	6.552 ***	−0.742 **	8.463	130.962 ***
Alaska	5.301 ***	11.434 ***	−1.456 ***	−37.840 *	−21.078
Arizona	1.601 *	1.331	−0.603 *	26.257	40.217
Arkansas	4.281 ***	7.639 ***	−1.803 ***	4.677	−0.267
California	1.23	2.052 ***	−1.255 ***	−9.454	−13.059
Colorado	3.544 ***	5.263 ***	−2.170 ***	13.922	56.646 **
Connecticut	3.274 ***	6.491 ***	−1.879 ***	77.685 ***	45.363 ***
Delaware	3.484 ***	4.456 ***	−0.999 ***	82.913 ***	92.635 ***
Florida	−0.901	−0.934	−0.146	22.509	29.847
Georgia	−0.274	−0.017	−0.698 **	27.353	23.036
Hawaii	−0.472	−2.238 ***	−0.941 ***	−14.283	48.593 ***
Idaho	3.446 ***	9.786 ***	−1.432 ***	20.483	−16.298
Illinois	1.804 **	3.054 ***	−1.452 ***	102.767 ***	79.879 **
Indiana	2.993 ***	5.724 ***	−1.606 ***	112.698 ***	14.385
Iowa	4.663 ***	5.876 ***	−1.721 ***	60.819 ***	65.506 ***
Kansas	2.129 ***	6.203 ***	−1.524 ***	60.459 ***	25.716
Kentucky	0.801	3.582 ***	−0.712 **	41.105 *	−40.937
Louisiana	−0.045	2.213 ***	−0.543 **	26.778 **	47.933 ***
Maine	8.739 ***	17.115 ***	−3.025 ***	−8.546	90.497 **
Maryland	1.886 ***	2.884 ***	−1.389 ***	45.462 ***	9.181
Massachusetts	1.435 **	2.210 ***	−1.650 ***	44.170 ***	114.490 ***
Michigan	7.414 ***	9.527 ***	−3.391 ***	94.356 ***	171.615 ***
Minnesota	2.953 ***	1.618 **	−1.798 ***	−28.276	6.562
Mississippi	2.670 ***	7.453 ***	−1.521 ***	−11.736	96.962 ***
Missouri	3.593 ***	4.524 ***	−1.628 ***	54.355 **	34.588
Montana	6.234 ***	14.457 ***	−1.740 ***	−31.103	42.468 *
Nebraska	2.717 ***	5.805 ***	−1.627 ***	60.033 **	40.155
Nevada	3.391 ***	3.997 ***	−1.738 ***	19.378	96.740 ***
New Hampshire	3.563 ***	6.551 ***	−1.549 ***	4.634	−45.906 **
New Jersey	4.421 ***	5.173 ***	−2.516 ***	121.265 ***	25.709
New Mexico	0.467	0.928	−0.174	66.458 **	92.059 ***
New York	4.704 ***	3.971 ***	−2.589 ***	−20.098	75.154 ***
North Carolina	0.833 *	3.730 ***	−0.758 ***	14.168	103.169 ***
North Dakota	6.482 ***	9.627 ***	−3.805 ***	102.288 ***	7.487
Ohio	3.293 ***	5.526 ***	−1.917 ***	66.207 ***	113.377 ***
Oklahoma	1.574 ***	4.082 ***	−1.122 ***	24.566 *	44.412 ***
Oregon	2.313 ***	2.855 ***	−0.822 ***	24.030 *	2.971
Pennsylvania	3.460 ***	3.112 ***	−1.757 ***	82.505 ***	37.785
Rhode Island	2.923 ***	4.760 ***	−1.601 ***	88.698 ***	103.377 ***
South Carolina	0.785	6.750 ***	−0.322	8.372	2.696
South Dakota	8.096 ***	18.190 ***	−2.568 ***	33.562 *	−3.449
Tennessee	2.179 ***	4.025 ***	−1.446 ***	−7.826	−14.521
Texas	0.925	2.018	−0.831	15.444	−47.087
Utah	3.788 ***	4.185 ***	−1.621 ***	−115.373 ***	−52.196
Vermont	4.603 ***	2.946 ***	−1.626 ***	−4.525	−72.788 **
Virginia	0.661	2.192 ***	−0.503	48.172 ***	25.314
Washington	2.700 ***	3.744 ***	−1.461 ***	−17.396	−27.258
West Virginia	3.937 ***	13.001 ***	−1.173 ***	4.823	12.985
Wisconsin	3.944 ***	6.343 ***	−1.662 ***	−28.834	−47.157 **
Wyoming	7.378 ***	14.447 ***	−2.097 ***	19.855 *	119.008 ***

## Appendix C. Counterfactual Analysis and Graphical Model Evaluation

The following regression results are based on fitting the models (3,4,5) to smoothed data (7-day moving average). Table A2 presents the regression estimates and Table A3 displays the estimated state-specific time trends. Fitted curves with and without policy are plotted in Figure A4 and Figure A5, which shows satisfactory results when compared to the actual observations. Again, the primary goal for fitting the models built in the main text to smoothed data was to see if the quality of the predicted curves would be improved (see the state-specific time trends subsection from the results section in the main text for more details).

**Table A2:** The impact of policy on COVID-19 cases, and mortality of COVID-19 (7-day average).

	Confirmed Case Growth Rate (3)	Death Case Growth Rate (4)
Stay-At-Home Order		
1 week	−2.473	2.859
2 weeks	−4.003 ***	−1.161
3 weeks	−3.894 **	−4.432 **
4 weeks	−4.129 **	−5.813 ***
One to two months	−2.849	−7.251 ***
More than 2 months	−2.941	−8.011 ***
Workplace Closure		
1 week	3.530 *	1.041
2 weeks	0.846	0.302
3 weeks	−0.695	0.73
4 weeks	−2.014	0.552
One to two months	−2.242	0.721
More than 2 months	−2.378	0.565
School Closure		
1 week	0.504	−1.042
2 weeks	0.287	2.334
3 weeks	−0.776	3.949
4 weeks	−0.943	4.413
One to two months	−2.156	4.403
More than 2 months	−2.234	3.626
Public Event Cancellation		
1 week	1.024	−1.124
2 weeks	−0.083	−1.053
3 weeks	−2.637	−1.191
4 weeks	−4.292	−2.3
One to two months	−6.542 *	−2.869
More than 2 months	−6.856 **	−3.031
Public Transport Closure		
1 week	−3.472	4.576
2 weeks	−2.933	7.351 **
3 weeks	−2.481	7.618 **
4 weeks	−3.924	7.114 **
One to two months	−5.108	6.106
More than 2 months	−4.958	6.548
International/National Travel Control		
1 week	−1.548	3.16
2 weeks	−3.283 **	4.018
3 weeks	−4.360 ***	3.476
4 weeks	−3.952 **	3.097
One to two months	−4.369 **	2.245
More than 2 months	−3.702	1.703
Public Information Campaign		
1 week	−5.535 ***	0
2 weeks	−11.682 ***	−2.354 *
3 weeks	−14.123 ***	−3.836
4 weeks	−12.535 ***	−4.564 *
One to two months	−13.490 ***	−5.092
More than 2 months	−14.926 ***	−5.109 *
Test growth rate	0.844 *	0.888
Confirmed growth rate		57.652 ***
constant	25.284 ***	8.39
r2	0.942	0.941

Notes: * *p* < 0.1, ** *p* < 0.05, *** *p* < 0.01. State-specific time trends corresponding to this table are shown in Table A3.

**Table A3:** State-specific time trends (as part of Table A2).

Time Trend	Confirmed Case Growth Rate (3)	Death Case Growth Rate (4)
Alabama	10.155	59.095 ***
Alaska	−32.622 **	−27.394 **
Arizona	13.227	26.447
Arkansas	36.154 **	35.957 ***
California	−10.765	8.219
Colorado	30.863 **	45.522 ***
Connecticut	87.160 ***	30.606 ***
Delaware	87.250 ***	−6.762
Florida	11.232	17.597
Georgia	22.545	23.376
Hawaii	−18.469	24.438 ***
Idaho	15.471	11.341
Illinois	89.965 ***	39.134 ***
Indiana	107.613 ***	−24.486
Iowa	65.835 ***	47.763 ***
Kansas	70.062 ***	−10.026
Kentucky	55.060 ***	−28.581
Louisiana	58.652 ***	26.203 **
Maine	−5.964	5.219
Maryland	67.261 ***	−12.463
Massachusetts	52.906 ***	60.880 ***
Michigan	108.931 ***	86.237 ***
Minnesota	−5.812	76.104 ***
Mississippi	7.858	59.597 ***
Missouri	45.380 ***	10.98
Montana	−36.921 **	−32.325 ***
Nebraska	75.301 ***	−11.472
Nevada	32.68	58.327 ***
New Hampshire	33.303 **	−4.022
New Jersey	126.371 ***	25.26
New Mexico	56.363 ***	39.292 ***
New York	16.115	58.550 ***
North Carolina	36.208 ***	78.614 ***
North Dakota	95.539 ***	9.175
Ohio	59.071 ***	68.370 ***
Oklahoma	21.958 *	51.080 ***
Oregon	25.486 **	18.069 **
Pennsylvania	93.768 ***	46.247 ***
Rhode Island	115.405 ***	12.364
South Carolina	12.68	−8.28
South Dakota	93.266 ***	−23.326 **
Tennessee	0.399	24.711
Texas	6.735	−14.084
Utah	−70.030 ***	6.727
Vermont	−2.488	−37.549 **
Virginia	44.177 ***	23.941 **
Washington	−25.989	−2.938
West Virginia	29.909 ***	81.529 ***
Wisconsin	−12.118	−2.851
Wyoming	26.938 **	148.983 ***
